# Austrian Heart Association and WHO Self-Care Guidelines. A qualitative study

**DOI:** 10.21542/gcsp.2024.42

**Published:** 2024-11-01

**Authors:** Wolfgang Mastnak

**Affiliations:** 1 Beijing Normal University, 19 Xinwai Ave, Beitaipingzhuang, Haidian District, Beijing, 100875, China; 2 European Academy of Sciences and Arts, Austria; 3 Austrian Heart Association, Innsbruck, Austria

## Abstract

**Background and Aim:** In its 2022 revision of self-care guidelines, the World Health Organization underlines the irreplaceable function of high-quality self-care and emphasises that not only research into self-care, but also the development of self-care-specific research methods have to be intensified. Accordingly, improvement of translational cardiological self-care is a key challenge faced by the Austrian Heart Association (Österreichischer Herzverband = ÖHV), which has been dedicated to life-long/long-term rehabilitation and health promotion of heart patients for more than 40 years. In line with the WHO call for self-care specific research, a study to identify related characteristics and desiderates was carried out with 30 regional managers at the ÖHV federal state conference [ÖHV Bundesländertagung] in 2023.

**Methods**: Through Mayring’s method of qualitative content analysis, raw data were regrouped into categories and frequencies/weights identified.

**Results**: Well-known benefits such as social inclusion, empathetic communication, mutual support, health sports and information were distinguished in a future-oriented manner, e.g., with regard to expansion of sports disciplines alongside more transparent adjustment to individual cardiorespiratory conditions.

**Conclusion**: Health policymakers are called to recognise the benefits of cardiac self-care and –  according to WHO suggestions – improve its integration within national health systems, standardised financial support included.

## Introduction

In numerous countries, cardiovascular-oriented self-care contributes considerably to holistic health improvements of cardiac patients and is often seen as an indispensable domain complementing professional cardiology – especially with regard to long-term rehabilitation. In Austria, the Austrian Heart Association has been contributing to this area of health care for over 40 years, continuously responding to changing requirements for long-term cardiovascular rehabilitation and corresponding research.

Research on cardiovascular self-care mirrors the broad spectrum of this complex domain in public health, recently with particular focus on heart failure related self-care. In general, self-care concerns individual styles of coping with illness^1^ as well as exercise programs and psychological support^2^. It relates to diversity in the sense of gender-specific cardiac self-care^3^ or an urban-rural divide in cardiovascular long-term rehabilitation offers^4^. It takes up traditional cardiac self-care topics such as personality-psychological adjustment after a heart attack^5^ or opens up new avenues such as the use of metacognitive therapy techniques to modulate anxiety and depression in cardiovascular diseases in self-care-specific group settings^6^.

These are also key topics of the Austrian Heart Association, which call for a still outstanding referential review of international research on cardiovascular self-care alongside implications on related standards. Given the marked heterogeneity of quantitative approaches in this area, meta-analyses are rather unlikely to be feasible. Systematic reviews, however, would not cause problems, but should go beyond conventional synoptic listing modes and thus provide a consistent construct with inherent cardio-rehabilitative logic.

The 2022 revision of the WHO guidelines on self-care interventions^7^ implies such a construct, but refers to the broad spectrum of all health-related self-care topics of supra-regional interest and concerns both countries with a highly developed health system and developing countries with an urgent need to catch up. In the introduction, Soumya Swaminathan, chief scientist of the World Health Organization, highlights (p. vii):

*Self-care interventions are playing a more prominent role in today’s world [...] The development of normative guidance from the World Health Organization (WHO) on self-care interventions acknowledges the important roles of individuals and communities in their own healthcare [...] We urgently need to identify innovative research methodologies to better understand self-care and how it fits into individual, community and national healthcare.*

**Figure 1.**
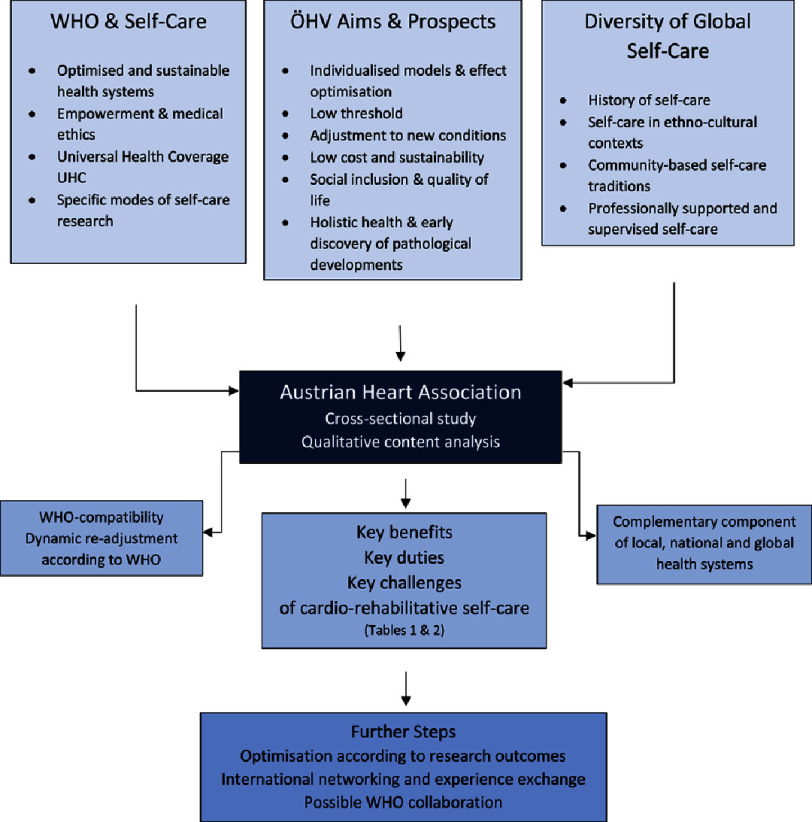
Austrian Heart Association & WHO Self-Care-Guidelines.

These demands are in line with key principles of the Austrian Heart Association, and – with regard to the WHO guidelines – following features have to be pointed out: the ÖHV activities are comprehensive and meant to be of lifelong sustainability; they strive for social justice and low-threshold participation; they are both self-help-based and oriented towards relevant cardio-rehabilitation research, thus meeting self-care-specific requirements of translational medicine (Figure 1).

Regarding international standards, public health compatible self-care is target-group oriented, science-based, and adapts dynamically to socio-pathogenic conditions. According to the World Health Organization, advanced self-care requires new research modes and meta-methodologies, which may considerably differ from standardised medical and health-scientific research designs despite certain overlaps and similarities. The Austrian Heart Association regards itself committed to these quality criteria that also form the subject of the present article.

Regarding the broad range of aims and aids of the Austrian Heart Association, WHO self-care guidelines require multiple interpretations that also related to the ‘Health Systems-Self-Care’ triad (p. xiv) *Self-Management–Self-Testing–Self-Awareness*. According to ÖHV-principles, these suggestions particularly concern (i) relevant physiological parameters such as blood pressure control, INR testing or BMI monitoring, (ii) early detection of recurrences alongside prevention of ‘heart panic’ or cardiovascular associated obsessive thought, (iii) group-based self-exploration of mental and/or psycho-affective problems, determination of their severity, and decision about possible subclinical support within ÖHV groups or encouragement to seek professional help, (iv) psychological and psychosocial self-regulation such as through mutual help based on relevant personal experience, and initiation of individual coping processes, (v) health-promoting lifestyles, including health sports for cardiorespiratory fitness, general skeletal-muscular mobility, and mental health alongside enhancement of neuroplasticity, (vi) interactive therapy planning with specialists, compliance and trust, as well as complementary competence for health promotion.

Self-care that complies with WHO guidelines can importantly reduce public health expenses and optimise inclusive and sustainable health systems, which is why countries are called upon to provide appropriate conditions. Since Austria is – just like Slovakia – a member of the United Nations and therewith represented in the World Health Assembly (WHA), regional and national health policies should regard suggestions such as expressed in chapter 2.4.5 of the WHO guidelines referred to:

*Health systems must also consider the potential savings that may result from earlier diagnosis and treatment due to self-care, and include these in the financial equation [...] Countries should review and, where necessary, revise laws, policies and regulations to ensure that quality self-care interventions are made widely available in the community, that they are also accessible to all without discrimination, through public, private and community-based health workers, and that they are acceptable to users.*

## Methods & study participants

The present study refers to clear-cut passages of the WHO self-care guidelines that emphasise the urgent need for innovative research and research methodology to better understand and develop self-help in the sense of individual, community-based and national healthcare.

Qualitative raw data of the present cross-sectional study were collected at the Austrian Heart Association’s annual conference from 4th to 6th of May 2023 in Radenci (Slovenia), including representatives of the ÖHV regional associations Burgenland, Carinthia, Lower Austria, Upper Austria, Styria, Tyrol (including Vorarlberg) and Vienna ( *n* = 30). Four to six answers were requested to each of the following key questions:

• *In your personal opinion, what is characteristic of the ÖHV work in your regional association?*
• *Which of your regional ÖHV association and/or district group activities are outstandingly good?*
• *What should your regional association definitely tackle?*
• *What can your regional association provide that the professional medical domain usually cannot?*
• *What are key topics that research into the ÖHV should address?*

The data collection was conducted on the 5th of May 2023 in the Congress Hall of the Health Centre (Zdravilišče) and Cardiology Clinic Radenci/Slovenia. The questions were presented to the entire group of participants in the form of a Powerpoint presentation along with a brief explanation that answers should be concise and truthful, and finally help improve personalised self-care based long-term cardiac rehabilitation. The participants had one hour to answer the questions on blank sheets of paper and were not allowed to speak to each other.

The following selected quotations (original in German language in square brackets) illustrates the raw data which were used to identify performance profiles and dynamic challenges of cardiac self-care:

***Knowledge transfer based on personal experience in intensive discussions*** *[Aufklärung durch Wissen aus eigener Erfahrung in intensiven Gesprächen];* ***mutual appreciation and consideration, no discrimination*** *[Gegenseitige Wertschätzung und Rücksichtnahme, keinerlei Diskriminierung];* ***regular hiking together in performance-matched groups*** *[Regelmäßiges gemeinsames Wandern in leistungshomogenen Gruppen];* ***recruiting younger members and improving the implementation of self-care in public health*** *[Akquirierung jüngerer Mitglieder und verbesserte Implementierung von Selbsthilfe im öffentlichen Gesundheitswesen];* ***Improving cooperation between self-help groups, physicians and representatives of other health professions, especially to promote health*** *[Verbesserung der Zusammenarbeit von Selbsthilfe, Medizinern und Vertretern weiterer Gesundheitsberufe, vor allem auch zur Förderung von Gesundheit];* ***improve public opinion on self-care, especially for prevention and those affected*** *[Meinungsbildung über Selbsthilfe in der Bevölkerung verbessern, besonders für Prävention und Betroffene];* ***research into savings in public health through self-care, *e.g.*, due to healthier lifestyles and positive influence on psychosocial values*** *[Erforschung der Einsparungen im öffentlichen Gesundheitswesen durch Selbsthilfe, z.B. durch gesündere Lebensstile und positiven Einfluss auf psychosoziale Werte].*

The collected raw data were analysed by means of Mayring’s qualitative content analysis. While the first screening process identified main categories and central topics, the second phase aimed at differentiation within the categories and contained calculation of quantitative clusters.

## Results

Table 1 (performance profile) and Table 2 (development profile) provide a pooled outline of the given qualitative content analysis. Numbers indicate cumulative responses, meaning that similar aspects in one and the same answer block were only counted once. The percentages indicate relative occurrences within the total. Although the present categories derive from critical content analysis and the epistemological attempt of adequate thematic grouping, also other categorisations and frameworks would have been possible. However, within the realm of Mayring’s qualitative content analysis this is not considered as shortcomings. From the perspective of philosophy of science, we may speak of a pragmatically oriented categorisations that rather provide cumulative tendencies than absolute analytic objectivity.

**Table 1:** Performance profile.

Topic	Characteristics	Frequency
Communication	Reliable contacts, confidential discussions, individual advice, supportive patient-physician communication style (lowering inhibition threshold), exchange of experiences (coping), contact point for those affected (including family caregivers), open discussions, tolerance of contradictory views, exchange with other (non-cardiovascular oriented) self-help groups, ideas forum, inspiration for innovation	20% (38)
Health sports	Hiking in performance-homogeneous groups, coronary gymnastics, regularity and sustainability, professional guidance, interactive (exchange of experience) optimisation, breadth of offers (e.g., swimming, cycling, dance)	17% (32)
Community	Sustainable social contacts, mutual appreciation, no discrimination, helpfulness, friendship, ‘extended family’, cultural-religious sensitivity (e.g., Christmas), feel-good factor, security, alternative settings (e.g., cardio care coffee), sustainability of care, group dynamics, team building (no ‘jealousies’)	16% (31)
Information	New medication options, microinvasive procedures, cardiovascular rehabilitation procedures, complex health promotion, comprehensibility & topicality, no lack of information about one’s own health compared to physicians, precautionary principles, explaining complex processes simply (but correctly), psycho-cardiology/psychological factors affecting the cardiovascular system, preoperative (self-care-specific) education, complex benefits of cardiac exercise and exercise therapy, factors (pathogenesis) of coronary diseases, receipt of the heart journals, heart day (‘Herztag’), information events in communities as an alternative to heart days	15% (28)
Public presence	Reputation and awareness in the public, cooperation with health policy, new media & media work in general, digitalisation	9% (16)
Mental hygiene	Mood stabilisation, resilience, ‘interception’ of risk dispositions, mindful dealing with worries, understanding mental hygiene in a systemic and holistic way, psychosocial inclusion, empathic support to cope with anxiety and/or depression (also in the context of loneliness), control of pathological personality change in those who become ill at an early stage, dealing with self-relevant and mental conflicts, using systemic group intelligence to solve problems, psychological improvement through sports, grief work	7% (13)
Lifestyle	Support for individual optimisation, nutrition, improvement of quality of life, positive everyday coping (re-adjustment), motivation for lifestyle changes, complex everyday-oriented support after rehabilitation	5% (10)
Prevention	Cardiovascular prevention, psychological prevention and mental health, individual prevention of recurrence, isolation and psychopathological consequences, identification of familial risk dispositions	5% (9)
Values	Readjustment of life and/or existence values, psychosocial ethics, reliable and stable ÖHV management, time for health as value in itself, positive reorientation of life, self-help competence, self-care related ‘self-sacrifice’ (pro/contra), recognition of self-care as part of political ethics	4% (8)
Assistance with authorities	Support with applications etc.	2% (3)

**Table 2:** Development profile.

Topic	Characteristics	Frequency
Research	Identification of ÖHV benefits, outcome research (e.g., reduction of early retirement), efficiency research, identification of new requirements, reviews of relevant cardiac self-care topics, intervention standards of the ÖHV, psychosocial self-help ethics, future orientation, interdisciplinarity (e.g., self-help & pharmacology), self-help under pandemic conditions, self-help under geographical-sociocultural local conditions, expansion of science-based self-help measures, holistic self-help, individual attitudes and attitudes towards illness, sustainability of self-help, financing of self-help, case series studies, gender perspectives of self-help, cardiac self-help & geriatrics, self-help & palliative medicine, individualised self-regulation (inspired by self-help), extended focus groups	30% (23)
Health policy & financing	Mindful communication with politicians, standardised funding, regular implementation in health care systems, politicians need to recognise the values of self-help, communication with similar associations and strategic transparency, efficient coordination with social insurance & health insurance companies	25% (19)
Member profile	Recruit younger members, increase membership, reactivate inactive members	25% (19)
Rehabilitation chain optimisation	Expansion of cooperation between inpatient rehabilitation and ÖHV, intensification of communication in the outpatient sector, systemic cooperation, comprehensive online networking of member	10% (8)
Administration	Standardisation & resort splitting within the association, motivation for ÖHV cooperation, marketing/health management	6% (5)
Training of ÖHV members	Telemedicine, artificial intelligence & cardiovascular rehabilitation, environmental factors (‘systemic cardiology’), self-help management, teamwork, public relations, sponsoring	4% (3)

## Discussion

The presented results mirror complex conditions of and dynamics within the participating regional sections of the Austrian Heart Association. Some item samples show considerable homogeneity throughout Austria, *e.g.*, high rating of community and communication, while heterogeneity within other categories demonstrates pronounced differences, *e.g.*, with regard to health policies and science orientation.

Thematic grouping of about 700 individual statements –  the qualifying word ‘about’ relates to partial vagueness and/or information overlaps – gave rise to two complementary areas: one highlighting characteristics and/or strengths of the respective regional association, the other emphasising desiderata or future prospects. Further differentiation led to clear-cut categorisation on which both tables were constructed. Quantitative information indicates the study participants’ responses, with obvious similarities only being counted once.

In terms of characteristics and strengths, communication, community, health sports and information were at the forefront, which is largely consistent with previous studies. With regard to community, sustainability and reliability, familiar atmosphere, friendship and social inclusion (in opposition to loneliness) were particularly emphasised, while the communication category revolved around empathetic interaction, exchange of information about coping with illness and life orientation, openness to confidential content and true willingness to listen. Easy accessibility, ‘having time’ and a long-term perspective were repeatedly regarded as essential quality features.

The information section refers both to the status quo, such as the ÖHV journals (Herzjournal), and to extensions, *e.g.*, self-care oriented training courses. Content differentiation and identification of key expectancies of ÖHV members will impact on the association’s internal information and training culture, while an overarching trend involves understandable knowledge transfer alongside precise coordination with individual medical conditions, health and rehabilitation profiles, coupled with practical implementation – in other words, genuine self-care oriented translationality.

For several decades health sports have markedly gained in importance in cardiac rehabilitation. Regarding exercise-based cardiac rehabilitation for coronary heart disease, a recent meta-analysis^8^ confirmed that participation in exercise-based cardiac rehabilitation by patients with coronary heart disease reduces cardiovascular mortality, recurrent cardiac events and hospitalisations. Additionally, the meta-analysis provided evidence supporting the improvement in health-related quality of life and highlighted the cost-effectiveness of exercise-based cardiac rehabilitation. Given that patients with coronary heart disease form a major group within the Austrian Heart Association, these findings are of high relevance for both rehabilitative and economical reasons. In patients with heart failure^9^, exercise training improves exercise capacity and quality of life, and reduces symptoms of depression as well as the risk for hospitalisation; findings which are fully compatible with the holistic bio-psycho-social approaches of the ÖHV. While a Cochrane study^10^ differentiated between home-based and centre-based cardiac rehabilitation, the Austrian Heart Association emphatically advocates integrative dynamic models. Widely complying with international studies on sports in cardiac rehabilitation, the present in-depth data analysis identified four further desiderata and challenges:

(i) expansion of the range of exercise options through different movement modes alongside the involvement of various sports disciplines such as Cardio Judo,
(ii) more appropriate use of local and/or geographical opportunities such as the Tyrolian mountains or the Pannonian Plain in Burgenland,
(iii) enhancement of individually relevant and highly specialised knowledge of health benefits, and
(iv) intensified efficiency evaluation within self-care groups.

These aspects are intertwined with the duality of lifelong/long-term rehabilitation and individualised holistic health promotion. However, the participants of the present study hardly mentioned issues of polymorbidity in terms of prevention and cross-diagnostic benefits of interventions, although these perspectives play an important role in the practice of the ÖHV – for example with regard to type II diabetes, progressive deterioration of musculoskeletal performance, cognitive decline or incipient dementia.

Complementary and partly interacting with health sports, mental hygiene – as far as addressed in this study – referred primarily to self-care specific coping with subclinical problems, help through authentic competence (in the sense of successful personal experience), ‘early diagnosis’, as well as empathetic support such as in grief work. Support from family caregivers appeared as important challenge within self-care areas as well. These topics partly overlap with key features of the category concerning lifestyle and communication.

Reviewing the current literature, studies on mental health in cardiac rehabilitation seem to be underrepresented. In line with the ÖHV’s strong focus on mental health hygiene, a Californian study^11^ pointed out that mental health conditions are associated with adverse cardiovascular outcomes in patients with ischemic heart disease. Although much of this risk can be attributed to poor health behaviours, ‘whether patients with mental health conditions are willing to participate in CR programs is unknown’. This is a main concern of the Austrian Heart Association, where mutual empathy, multifaceted interaction and social inclusion proved to importantly contribute to adherence and sustainability.

Some participants of the present study expressed harsh criticism about deficient health policy in several federal states of Austria (Bundesländer), particularly pointing out that health politicians apparently (i) have little knowledge of the benefits of high quality self-care, (ii) are not familiar with relevant documents of the World Health Organization, (iii) are not aware that WHO guidelines also apply to Austria, and (iv) have no competent familiarity with dynamic-systemic processes – particularly in the sense of Complexity Sciences and Health Care^12^ – in health care systems. Study participants requested more respectful treatment of service providers, who achieve high effects at low cost. This also applies to suggestions made about optimising a sustainable cardiovascular rehabilitation chain.

Consistent with WHO suggestions, innovative statements related to self-care specific research. According to the ‘Development Profile’ table, a broad spectrum of topics is meant to initiate further measures within the ÖHV – and questions about research funding and research cooperation come into play. This also relates to international activities that are in line with WHO paradigms to optimise health promotion under local conditions while optimising universal health coverage in a global sense.

How outcomes of the present qualitative research may globally apply to cardiac self-care involves several critical issues such as (i) the necessary distinction between conventional research designs of evidence based medicine and novel self-care typical research models, (ii) similarity and diversity of self-care groups and organisations, and (iii) dissimilar sociocultural, public health and geographical conditions. By way of illustration, a systematic review and meta-analysis protocol^13^ pointed out that heart failure oriented self-care interventions are recommended for preventing and detecting exacerbations, improving symptom management and preventing hospitalisations, although little were known about the overall effectiveness of heart failure self-care programmes and which types of interventions would show the greatest improvement. Consequently, the authors planned to synthesise and compare outcomes from controlled trials to strengthen heart failure self-care among adults. However, regarding complex effects of multidimensional self-care programmes, philosophy of science raises the question whether mono-dimensional controlled trials or complexity science that represents dynamic systems were more appropriate to tackle these questions. In this context, the WHO recommended to consider novel self-care typical research designs. The Austrian Health Association (section Tyrol) intends to go in for this urgent interdisciplinary meta-theoretical challenge.

From an international point of view, cardiac self-help is diverse, and characteristics differ from each other. While the Austrian Heart Association is an umbrella institution housing the formally independent heart associations of the federal states, *e.g.*, the Tyrolian Heart Association, the German Heart Foundation [Deutsche Herzstiftung] provides about 6000 heart groups [Herzgruppen], which are guided by physicians and differ to some extent from genuine cardiac self-care^14^. In the UK, the British Heart Foundation runs support groups^15^, and members of the Fudan University in Shanghai are discussing how to enhance cardiac self-care in the People’s Republic of China, and how related programmes may respect both complex cardiac rehabilitation and cultural conditions, *e.g.*, within ethnic groups living in China, from the Uighurs in the Northwest to the Hmong and Wa people in the South. Considering this very heterogeneous status quo, improved networking among cardiac self-care organisations may greatly advance the global situation.

In the developmental profile area (cf. Table 2) research is in pole position, and the Tyrolian Heart Association focuses particularly on five domains, (i) cross-cultural and culturally sensitive cardiac self-care and long-term rehabilitation. Intensified international collaboration is intended, *e.g.*, to explore the advantages of mountain regions for cardiac self-care sports, (ii) novel research designs that are tailored to self-care specific characteristics and can also be carried out by self-care groups. These meta-theoretical approaches importantly involve philosophy of science and theories of truth^16^, and contribute to medical epistemology and complexity science, (iii) research on optimised self-care models that are tailored to changing pathogenic and public health dynamics such as fighting social isolation and making telehealth ^17^ more accessible, (iv) reliable estimates of savings in the healthcare sector through low-cost self-care activities, and (v) identification of genuine characteristics and benefits of cardiac self-care, as well as its (irreplaceable) complementarity to cardiological interventions, such as support of empowerment, self-actualisation and anchoring healthy behaviour in daily lifestyles. These domains, which are markedly inspired by the outcomes of the present study, determine future research directions of the Tyrolian Heart Association, and encourage international collaboration as well.

### Limitations of current study

i. the raw data are based on experiences and opinions of representatives of the Austrian Heart Association. Although they are permanently confronted with both self-care related health policies and multifaceted concerns of heart patients, there is a clear need for further empirical studies with heart patients participating in cardiac self-care groups;
ii. the study was conducted in Austria, a country with advanced clinical facilities and public health alongside distinct health-relevant sociocultural conditions, *e.g.*, the Alpine regions and folk music with health promoting character. Further studies have to focus on cross-cultural transferability of the present outcomes and how to re-adjust findings according to different conditions;
iii. regrouping data according to Mayring’s qualitative content analysis requires heuristic considerations and is never ‘absolute’. This very limitation involves philosophy of science and correspondence theory of truth, as well as meta-methology of medical research.

## Conflict of Interest

The author is – until end of 2024 –  president of the Austrian Heart Association.
